# Predictors of operative ischemic cerebrovascular complications in skull base tumor resections: Experience in low-resource setting

**DOI:** 10.1093/nop/npae063

**Published:** 2024-07-15

**Authors:** Mestet Yibeltal Shiferaw, Abat Sahlu Baleh, Abel Gizaw, Tsegazeab Laeke Teklemariam, Abenezer Tirsit Aklilu, Atalel Fentahun Awedew, Denekew Tenaw Anley, Bereket Hailu Mekuria, Ermias Fikiru Yesuf, Mengistu Ayele Yigzaw, Henok Teshome Molla, Alemu Adise Mldie, Mekides Musie Awano, Abraham Teym

**Affiliations:** Department of Surgery, Neurosurgery Unit, Debre Tabor University, Debre Tabor, Ethiopia; Department of Surgery, Neurosurgery Unit, Addis Ababa University, Addis Ababa, Ethiopia; Department of Surgery, Neurosurgery Unit, Addis Ababa University, Addis Ababa, Ethiopia; Department of Surgery, Neurosurgery Unit, Addis Ababa University, Addis Ababa, Ethiopia; Department of Surgery, Neurosurgery Unit, Addis Ababa University, Addis Ababa, Ethiopia; Department of Surgery, Debre Tabor University, Debre Tabor, Ethiopia; Department of Public Health, College of Health Sciences, Debre Tabor University, Debre Tabor, Ethiopia; Department of Surgery, Neurosurgery Unit, Addis Ababa University, Addis Ababa, Ethiopia; Department of Surgery, Neurosurgery Unit, Debre Birhan University, Debre Birhan, Ethiopia; Department of Surgery, Neurosurgery Unit, Hawassa University, Hawassa, Ethiopia; Department of Surgery, Neurosurgery Unit, Addis Ababa University, Addis Ababa, Ethiopia; Department of Surgery, Neurosurgery Unit, Jimma University, Jimma, Ethiopia; Department of Surgery, Neurosurgery Unit, Addis Ababa University, Addis Ababa, Ethiopia; Department of Environmental Health, College of Health Sciences, Debre Markos University, Debre Markos, Ethiopia

**Keywords:** cerebrovascular complications, iatrogenic injury, resection, skull base tumor, vasospasm

## Abstract

**Background:**

Ischemic cerebrovascular complications following skull base tumor resections remain a significant factor impacting both short-term and long-term patient outcomes. This study aims to improve risk stratification, surgical decision-making, and postoperative care protocols.

**Methods:**

A retrospective cohort study on predictors of ischemic cerebrovascular complications among patients who underwent skull base tumor resection was conducted at 2 high-volume neurosurgical centers in Ethiopia from 2018 to 2023. Binary logistic analysis was performed to see the association of each predictor variable.

**Results:**

The study included 266 patients, with 65.5% being female. The median age and tumor size were 37 (± IQR = 17) years and 4.9 cm (± IQR 1.5), respectively. Ischemic cerebrovascular complications occurred in 19.9% of patients. Middle cranial fossa tumors and tumors spanning both anterior and middle cranial fossa (AOR = 6.75, 95% CI: 1.66–27.54, *P* < .008), grades 3–5 vascular encasement (AOR = 5.04, 95% CI: 1.79–14.12, *P* < .002), near-total resection and gross total resection (AOR = 2.89, 95% CI: 1.01–8.24, *P* < .048), and difficult hemostasis (AOR = 9.37, 95% CI: 3.19–27.52, *P* < .000) were significantly associated with iatrogenic vascular injury. Subarachnoid hemorrhage had a statistically significant association with vasospasm (AOR = 12.27, 95% CI: 1.99–75.37, *P* = .007).

**Conclusions:**

Surgery-related ischemic cerebrovascular complications are common. Thorough perioperative risk stratification and proactive treatment planning are crucial to mitigate vascular insults associated with it. In low-resource settings, neurosurgical services are provided without advanced instruments, leading to more complications. Therefore, it is important to focus on improving neurosurgical setup to enhance patient outcomes.

Skull base tumors represent a complex array of neoplasms originating from the intricate skull base region.^1^ The anatomical complexity of the skull base, which contains vital neurovascular structures like major arteries, veins, and cranial nerves, poses a challenge during surgical interventions.^1–7^ Despite advancements in surgical techniques and perioperative care, the occurrence of ischemic cerebrovascular complications following skull base tumor resections remains a significant factor, impacting both short-term and long-term patient outcomes.^1,8–10^ Surgery-related ischemic cerebrovascular complications comprise a spectrum of ischemic insults that includes cerebral vasospasm, delayed cerebral ischemia (DCI), vascular thrombosis, pseudo-aneurysms, iatrogenic vascular injuries, and strokes.^11^

A myriad of factors, such as tumor-specific features, surgical approaches, and intraoperative variables, potentially contribute to the risk of ischemic cerebrovascular complications after skull base tumor resections. The available data on the topic are primarily derived from case reports and series, with a predominant contribution from otolaryngological surgeons. The absence of comprehensive studies in this domain from neurosurgical centers has hindered the identification of potential modifiable risk factors. Consequently, understanding the predictive factors related to cerebrovascular complications is crucial.^3,11–17^

Skull-base neurosurgical procedures have been practiced in Ethiopia since 2010 at 2 of the largest neurosurgical teaching affiliate hospitals of Addis Ababa University—The Black Lion Hospital and Myungsung Christian Medical Hospital (MCM). Although both centers have one of the highest case volumes in the nation, the actual burden of skull base lesions and factors associated with ischemic cerebrovascular complications in Ethiopia have not been investigated. Similarly, there is insufficient comprehensive study regarding the occurrence and contributing factors of ischemic insults probably because of the complexity and wide variety of the skull base tumors along with the different surgical approaches and corridors used. Thus, there is a need for studies that provide a robust foundation for risk stratification, refined surgical decision-making, and tailored postoperative care protocols that guide prognostication and patient care optimization. This multi-centric retrospective cohort study aimed to elucidate the predictors associated with ischemic cerebrovascular complications and their subsequent influence on clinical outcomes among patients undergoing skull base tumor resection.

## Methods

A multi-centric retrospective cohort study was conducted on the occurrence and predictors of ischemic cerebrovascular complications among patients who underwent skull base tumor resection from 2018 to 2023. The study was conducted at 2 of the largest teaching hospitals of Addis Ababa University—Black Lion Hospital and Myungsung Christian Medical Hospital (MCM). The study cohort was comprised of patients undergoing anterior and middle cranial fossa tumor resection. All patients who had preoperative, intraoperative, and postoperative clinical and image data (CT scan, MRI, or CT angiography) in the electronic patient registry and imaging database of both neurosurgical centers were included in the study. Patients with absent or incomplete registry data and follow-up imaging were excluded. Ethical approval was obtained from the institutional review board (IRB) of Addis Ababa University.

### Surgical Techniques and Resources

Neurosurgeons with experience ranging from 1 to 14 years have performed procedures on all types of skull base tumors, regardless of complexity, without a specific area of focused practice in our setting.

While the use of surgical adjuncts like image guidance and intraoperative localization of vascular structures (eg, doppler, intravascular fluorophores) are understandable of considerable significance, our setup has never used this surgical adjunct in the effort to localize the major vessel in relation to the tumors due to unavailability of gadgets and limited experience of the operating neurosurgeons in our setting. Preoperative CT-angiography usually was obtained for tumors in high-risk locations (eg, clinoidal meningioma) to assess the vessel encasement and its relation to the tumor.

There was no adjunctive stereotactic radiosurgery at all and the use of conventional radiotherapy is also limited in most instances, making surgery the mainstay of treatment in our setting. To reduce the vascularity of the tumor extradural dissection of the tumor has been practiced by most of the neurosurgeons in our setup. However, there has never been the utilization of angiographic (endovascular) embolization to reduce the vascularity of the tumor due to the absence of endovascular intervention angio suit. In addition, endovascular interventions like mechanical and chemical angioplasty have never been utilized for patients with inadvertent iatrogenic vascular injury and vasospasm.

Mannitol has been the routine standard agent for achieving brain relaxation during brain tumor surgery even if it sometimes is not as effective in causing brain relaxation, especially in huge tumors. However, there has been no standard protocol for using the lumbar drain for huge tumors in our setting. It was practiced to a handful of patients by some neurosurgeons and the degree of brain relaxation achieved was satisfactory. Hence, there has been a growing interest in using it despite the lack of a lumbar drain limiting its frequent use. The use of an external ventricular drain for brain relaxation to supra-tentorial tumors is non-existent in our setting.

The mode of tumor resection has been by using microsurgical techniques using micro scissors, bipolar cautery, etc. and there has never been the use of a surgical ultrasonic tissue aspirator (CUSA) due to unavailability. As a result, it has been customary to take long operating hours for, a high incidence of brain contusion, brain edema, and vascular avulsions during procedures. These all contribute to difficult surgery and a high incidence of vascular injury directly or indirectly.

Trans-sphenoidal (TSS) procedures have been performed using an endoscope, microscope, and sometimes both. However, mostly microscopic TSS has been performed. All neurosurgeons themselves perform the exposure part of TSS and ENT surgeons have never assisted in the exposure part of the TSS. Similarly, the skull base reconstruction commonly utilizes multi-layered tissue reconstruction, including fat, naso-septal flap, osteo-cartilage tissue, and hemostatic agents like gel foam &/surgicel.

The extent of resection was determined by using follow-up postoperative imaging that was obtained within the first 48 hours of the surgery; and was not based on the surgeon’s impression. In addition, the extent of resection for all patients was cross-checked with the neuroradiologist’s comment that has been available in the image database of our setup.

### Outcome Measure (Dependent Variable)

The outcome variable was a composite variable of ischemic cerebrovascular complications (Yes/No). The composite variable included angiographic cerebral vasospasm, clinical cerebral vasospasms or DCI, DCI-related cerebral infarction, ischemic stroke, cerebral venous or arterial thrombosis, iatrogenic vascular injury, and pseudo-aneurysm. The diagnosis of ischemic cerebral vascular complications was confirmed by consistent postoperative follow-up imaging findings suggestive of it and a set of standard clinical criteria as detailed in the operational definition. More precisely, the composite outcome measure was summarized as vasospasm and iatrogenic vascular injury so that separate analysis could be possible.

### Predictor (Independent) Variables

The study included independent or predictor variables that possibly affect the occurrence of ischemic cerebrovascular complications (mainly cerebral vasospasm and iatrogenic vascular injury). This includes sociodemographic factors (comorbidity, preoperative Karnofsky performance status [KPS], age, sex, and prior surgery), preoperative imaging factors (tumor type, location of tumor, size of tumor, radiation/embolization, and grade of vessel encasement), Intraoperative surgical factors (surgical approach, brain edema, brain contusion, experience of surgeon, estimated blood loss, difficulty of securing hemostasis), and postoperative factors (follow-up imaging, extent of resection, tumor bed hematomas, hydrocephalus, pneumocephalus, contusion, brain edema, cerebral infarction, etc.).

### Operational Definitions

Iatrogenic vascular injuries or ischemic stroke during tumor excision were diagnosed by the control postoperative CT or MRI images obtained within the first 24–48 hours after surgery.

The **diagnosis of vasospasm** in our setup has been challenging as Trans Cranial Doppler ultrasound is unavailable and angiography is expensive. Consequently, the diagnosis of clinical vasospasm (DCI) was considered retrospectively after 3–4 postoperative days in patients who had a normal brain CT (mostly used) within the first 1–2 days of the postoperative period, as defined by the international expert panel for SAH Research 2010 guideline.^18^

**DCI** is defined as a new focal neurologic deficit lasting for at least 1 hour (such as hemiparesis, aphasia, apraxia, hemianopia, or neglect), or a decrease of at least 2 points on the Glasgow Coma Scale, which is not apparent immediately after tumor resection and cannot be attributed to other causes (HCP, re-bleeding, electrolyte disorders, seizures, and hypoxia) OR those with examinations too poor to clinically detect symptoms.^18^

**DCI-related cerebral infarction** is defined according to the international expert panel for SAH Research 2010 as the diagnosis of cerebral infarction performed by either a brain CT or MR scan within 6 weeks after SAH, or on the latest CT or MRI scan made before death within 6 weeks, or proven at autopsy, not present on the CT or MRI scan between 24 and 48 hours after early tumor resection/aneurysm occlusion, and not attributable to other causes, such as direct vessel injury during tumor resection/surgical clipping or endovascular treatment.^18^

**Cerebral vessel thrombosis** (arterial, veins, and venous sinus) was diagnosed when CT, CTA/MRA and MRV confirmed the presence of thrombus in suspected vessels.

The **extent of resections** was assessed using 2D methods rather than volumetric assessment, with the extent of resection classified as follows:

- Subtotal resection (STR): resection of < 95% of the tumor.
- Near total resection (NTR): resection of ≥ 95% of the tumor.
- Gross total resection (GTR): resection of 100% of the tumor.

The **grade of vascular encasement** by the tumor was determined based on circumferential involvement:—Grade 0: no involvement—Grade 1: 1°–89°—Grade 2: 90°–179°—Grade 3: 180°–269°—Grade 4: 270°–359°—Grade 5: 360°.

A **challenge in achieving hemostasis** refers to the technical difficulties encountered intra-operatively as documented in the operation note by the operating surgeon, and not challenges due to coagulopathy or failure to secure hemostasis at the end of the procedure.

### Data Collection and Analysis

A data collection tool was developed after reviewing previously published studies. Data was collected by the neurosurgeons to ensure data quality. The data sources were patient electronic records, imaging databases, and follow-up phone calls. Accurate diagnosis of the spectrum of ischemic cerebral complications was confirmed by experienced neuro-radiologists’ comments. Data analysis was conducted using SPSS version 25. Categorical variables were expressed in frequencies and percentages and compared using Chi-square or Fischer-Exact tests. Normal distribution tests were performed for continuous data using histograms and Shapiro–Wilk tests. Univariable analysis was done to assess the association of each predictor variable with ischemic cerebral vascular complications. Variables with a *P*-value of < .25 in the univariate analysis were entered into the multivariable analysis. The statistical significance was declared at a *P*-value < .05. The measure of association was the odds ratio or adjusted odds ratio with 95% confidence.

## Results

### Patient Demographic Characteristics

In a retrospective cohort of surgically treated 266 patients with skull base tumors, the median age of the patients was 37 (± IQR = 17) years, indicating that the average age of the study population was relatively young. Additionally, the majority of patients were female, accounting for 65% of the cohort. This gender distribution suggests a potential gender-based difference in the incidence or presentation of skull base tumors. Around 15.8% of patients had comorbidity. The comorbidities assessed include hypertension, diabetes mellitus, HIV, bronchial asthma, chronic kidney, ischemic chronic disease, congestive heart disease, chronic obstructive pulmonary disease, and prior ischemic stroke. The presence or absence of comorbidity wasn’t shown to have a statistically significant association with the incidence of vascular injury and vasospasm.

Similarly, 95.9% of patients who underwent skull base tumor resection had a Karnofsky Performance Status (KPS) of more than 70%, indicating a relatively good functional status in the majority of these patients (Table 1).

**Table 1.** Demographic Characteristics, Tumor Characteristics, and Clinical Presentation of Patients With Skull Base Tumor Resection in a Large Volume Neurosurgical Center, Ethiopia (*N* = 266)

Item	Variables	Frequency	Percent
Sex	Female	169	63.5
Age	Below 40	144	54.1
	above 40	122	45.9
Comorbidity	Yes	42	15.8
KPS	<70	11	4.1
	≥70	255	95.9
Redo surgery	Yes	29	10.9
Location	Middle cranial fossa	211	79.3
	Anterior cranial fossa	47	17.7
	Both	8	3
Tumors	Meningioma	182	68.4
	Pituitary macroadenoma	51	19.2
	Craniopharyngioma	24	9.0
	Other	9	3.38
Epicenter of meningioma (*N* = 182)	Enplaque meningioma	2	0.8
	Lateral &/middle sphenoid wing meningioma	29	10.9
	Middle fossa meningioma	1	0.4
	Clinoidal/medial SWM	40	15.0
	Olfactory groove meningioma	31	11.7
	Planum sphenoidale meningioma	17	6.4
	Sheno-orbital meningioma with/ without cavernous sinus extension	12	4.5
	TSM	50	18.8
Vessel encasement	Grade 0	49	18.4
	Grade 1	81	30.5
	Grade 2	61	22.9
	Grade 3	43	16.2
	Grade 4	19	7.1
	Grade 5	13	4.9
Preoperative hydrocephalus	Yes	25	9.4
Preoperative edema	Yes	123	46.2
Size	<4 cm	91	34.2
	≥4cm	175	65.8
Midline shift	<5mm	209	78.6
	≥5mm	57	21.4
Clinical presentation	Headache	228	85.7
	Weakness	24	9.0
	Seizure	44	16.5
	Sphincter dysfunction	24	9.0
	Vision impairment	219	82.3
	Smell impairment	27	10.2
	Impaired level of consciousness	30	11.2

Grade of tumor encasement was determined by the circumferential involvement as follows: Grade 0 (no involvement), grade 1 (1°–89°), grade 2 (90°–179°), grade 3 (180°–269°), grade 4 (270°– 359°), and grade 5 (360°).

### Clinical Profile

Headache and loss of vision were the most common clinical presentations among patients with skull base tumors, accounting for 85.7% and 82.7% of cases, respectively. The mean duration of symptoms at presentation was 17.3 months (±11.1) with minimum and maximum duration of symptoms being 1 and 84 months, respectively. Visual disturbances were common in our study due to the proximity of critical structures involved in vision, such as the optic nerves and chiasm with skull base tumors. The study included all anterior and middle cranial fossa base tumors, with 79.3% arising from the middle cranial fossa and the remaining tumors originating from the anterior cranial fossa. Meningioma was the most common type of tumor, accounting for 68.8% of cases, with the tuberculum sellae being the most common epicenter of these tumors. The median size of the tumor in this study was 4.9 cm (±IQR 1.5), indicating that the majority of tumors were of large size (Table 1).

The most common surgical procedure performed for those tumors was pterional craniotomy, which accounted for 81.6% of cases while 18.4% of patients underwent TSS. It was found that 22.6% of the 266 patients who had their masses resected had a mass effect on follow-up imaging after the procedure, and 6.8% of them had brain swelling during the procedure. The most common postoperative bleeding complications were intracerebral hemorrhage (ICH) at 9.2%, subarachnoid hemorrhage (SAH) at 8.6%, and acute subdural hematoma (ASDH) at 2.6%. Pneumocephalus occurred in 9.8% of cases and a variable extent brain contusion in 35.3% (Table 2).

**Table 2.** Intraoperative and Postoperative Factors in Patients With Skull Base Brain Tumor Resection in a Large Volume Neurosurgical Center, Ethiopia (*N* = 266)

Item	Variables	Frequency	Percent
Procedure	Craniotomy	217	81.6
	Trans-sphenoidal surgery	49	18.4
Intra-op factors	Brain contusion	83	31.2
	Brain Swelling	18	6.8
	Hemostatic agent use	201	75.6
	Difficult hemostasis	37	13.9
Estimated blood loss (EBL)	<1L	178	66.9
	1–2L	73	27.4
	≥2L	15	5.6
Extent of resection	Near total (NTR) or gross total (GTR) resection	171	64.3
	Subtotal resection (STR)	95	35.7
Postop changes on Control images	Subarachnoid hemorrhage (SAH)	23	8.6
	Acute subdural hematoma (ASDH)	7	2.6
	Intracerebral hemorrhage (ICH)	51	19.2
	Intraventricular hemorrhage (IVH)	25	9.4
	Epidural hematoma (EDH)	5	1.9
	Pneumocephalus	24	9.0
	Mass effect on control CT	60	22.6
Ischemic cerebrovascular complications	Iatrogenic Injury	42	15.8
	Vasospasm	11	4.1
Ischemic Vascular territories (*N* = 53)	Anterior cerebral artery (ACA)	8	15.09
	Basilar artery	1	1.51
	Major Cortical artery	2	3.77
	Artery of Heubner	2	3.77
	Internal carotid artery (ICA)	15	28.30
	Middle cerebral artery (MCA)	12	22.64
	Multiple artery territory	9	16.98
	Perforators	4	7.94
WHO grade of the tumor	1	251	94.4
	2	10	3.8
	4	5	1.8
CSF leak during trans-sphenoidal surgery	No	45	88.2
	Yes	6	11.8
In hospital mortality		56	21.1

An ischemic cerebrovascular insult occurred in 19.9% of patients; of these, 20.7% were related to cerebral vasospasm and 79.3% were iatrogenic vascular injuries. Remarkably, 79.2% of individuals impacted by vascular insults were female. Among patients with vascular insults, 79.3% had procedures for meningioma, 7.5% had procedures for pituitary tumors and the rest had other skull base brain tumors. Regretfully, a significant portion of patients who suffered from vascular insults—79.2%—died while they were hospitalized. It is indeed concerning that among the patients who experienced mortality after skull base mass resection; a significant proportion had cerebrovascular ischemic insults. Accordingly, the internal carotid artery, middle cerebral artery (MCA), and anterior cerebral artery were impacted in 28.3%, 20.8%, and 15.1% of cases, respectively, among patients with ischemic vascular insults during skull base tumor resection. The complexity of vascular complications in patients undergoing skull mass resection surgeries was further highlighted by the fact that multiple arterial territories were involved in 24.5% of cases (Table 2).

Looking into the WHO grade of tumors, 94.4 % of them were WHO grade 1 while the remaining 3.8 % and 1.9% were WHO grade 2 and 4, respectively.

Overall, there was an 11.8% (6/51) CSF leak incidence among patients who underwent TSS. Excluding other hospital-acquired infections among patients who underwent TSS, 9.8 % (5/51) of them developed meningitis and were treated with antibiotics and improved.

Indeed, iatrogenic vascular injury during resection of a skull base tumor is a catastrophic event that can result in a considerable degree of morbidity and mortality. To enhance patient outcomes and lower the likelihood of these complications, it is essential to identify the factors linked to the incidence of iatrogenic vascular insult. Preoperative and intraoperative factors were assessed in our analysis to ascertain their correlation with iatrogenic vascular injury.

Numerous factors were investigated to be associated with iatrogenic vascular injury during skull base tumor resection before controlling for several variables. These included the following: surgical technique, intraoperative brain contusion, sex, comorbidity, tumor location, tumor size, midline shift, vascular encasement grade, tumor type, and hemostasis challenges. These factors demonstrated a significant association with iatrogenic vascular injury before statistical adjustment. After adjusting for multiple variables using binary logistic regression analysis, several factors were shown to have a statistically significant association with iatrogenic cerebrovascular injury following skull base tumor resection. These factors, such as tumor location (AOR = 6.75, 95% CI: 1.66–27.54, *P* < .008), high-grade encasement (grades 3–5; AOR = 5.04, 95% CI: 1.79–14.12, *P* < .002), extent of resection (near-total resection or GTR; AOR = 2.89, 95% CI: 1.01–8.24, *P* < .048), and challenges in achieving hemostasis (AOR = 9.37, 95% CI: 3.19–27.52, *P* < .000), were identified as significant predictors of iatrogenic vascular injury during skull base tumor resection (Table 3). All procedures were performed by neurosurgeons with different years of experience that range from 1 year to 14 years. There was no statistically significant difference in the overall incidence of ischemic cerebrovascular complications according to this cohort.

**Table 3.** Univariate and Multivariate Analysis Results on Factors Associated With Iatrogenic Vascular Injuries in Patients With Skull Base Brain Tumor Resection in a Large Volume Neurosurgical Centers, Ethiopia (*N* = 266)

Item	Variables	Iatrogenic vascular injury		Uni-variate analysis		Multi-variate analysis	
				COR (95%CI)	*P*-value	AOR (95%CI)	*P*-value
		Yes	No				
Sex	Female	35	134	3.36 (1.43, 7.89)	.005	2.49 (0.79, 7.77)	.117
	Male	7	90	1		1	
Comorbidity	Yes	4	38	0.52 (0.17, 1.52)	.232	0.64 (0.19, 2.18)	.478
	No	38	186	1		1	
Location	Middle and / both anterior & middle cranial fossa	38	181	2.26 (0.76,6.66)	.141	6.75 (1.66, 27.54)	.008
	Anterior cranial fossa	4	43	1		1	
Size of tumor (cm)	Below 4cm	5	86	1		1	
	>/= 4 cm	37	138	4.61 (1.75, 12.19)	.002	1.87 (0.57, 6.24)	.304
Midline shift	Less than 5mm	27	182	1		1	
	>/= 5mm	15	42	2.41 (1.18, 4.92)	.016	0.81 (0.30, 2.18)	.680
Vascular encasement	Grade 0-2	6	124	1		1	
	Grade 3-5	36	100	7.44 (3.01, 18.36)	.000	5.04 (1.79, 14.12)	.002
Procedure	Craniotomy	40	177	5.31 (1.24, 22.78)	.025	1.34 (0.17, 10.32)	.782
	TSS	2	47	1		1	
Intraoperative brain contusion	No	165	18	1		1	
	Yes	59	24	3.73 (1.89, 7.36)	.000	1.52 (0.54, 4.25)	.430
Extent of resection	NTR or GTR	32	139	1.96 (0.91, 4.18)	.083	2.89 (1.01, 8.24)	.048
	STR	10	85	1		1	
Tumor	Meningioma	38	144	5.28 (1.82, 15.32)	.002	2.66 (0.49, 14.56)	.260
	Non-meningioma	4	80	1		1	
Difficult hemostasis	Yes	20	17	11.07 (5.07, 24.19)	.000	9.37 (3.19, 27.52)	.000
	No	22	207	1		1	

### Illustrative Case for Iatrogenic Vascular Injury During Skull Base Tumor Resection

**Figure 1.**
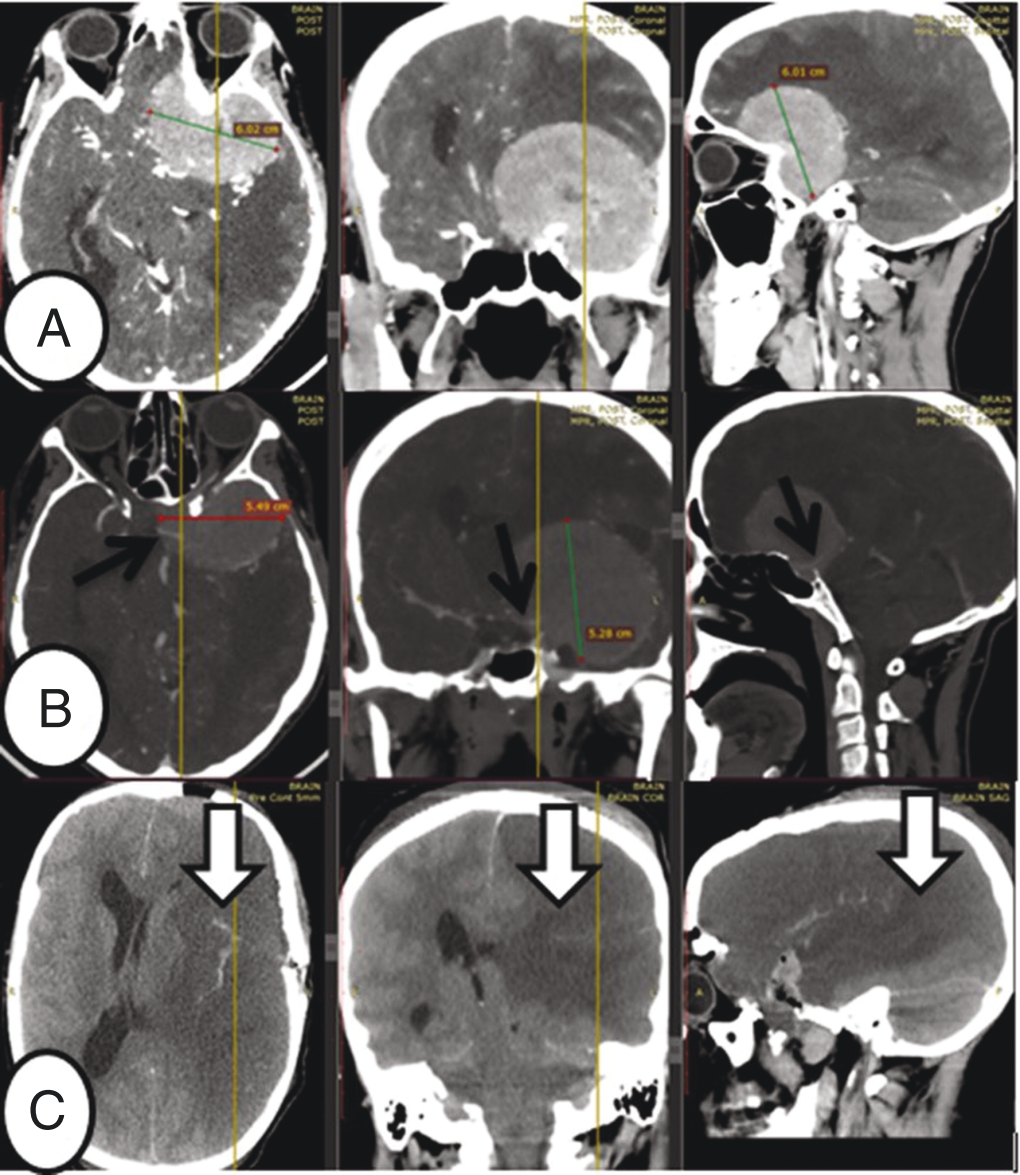
This figure depicts the image of a patient who was diagnosed to have left cerebral infarction secondary to iatrogenic middle cerebral artery (MCA) injury during surgery for a left-side clinoidal meningioma. The image in (A) was a preoperative CT scan with contrast of a patient and showed giant left clinoidal meningioma. The arrow in (B) depicted the CT angiography of a patient who had a 360-degree encasement of the MCA by the tumor. Follow-up CT was obtained for the same patient within 6 hours of the completion of the surgery. The down-pointing arrows as depicted in (C) showed left MCA infarction due to iatrogenic injury of MCA.

A 48-year-old female patient presented with a complaint of left visual loss of 13 months and headache of 3 years. Otherwise, she did not have seizures, weakness, and sphincter dysfunction. Objectively, the patient had no light perception on the left side. No other neurologic and other system findings. On imaging, there was a left giant-sized avidly enhancing clinoidal meningioma encasing the major vascular structures (Figure 1A and B). GTR of the tumor was achieved and the patient was transferred to ICU. Her GCS was low in the immediate postoperative period. Hence, a follow-up CT was obtained for the same patient within 6 hours of the completion of the surgery and it showed MCA infarction (Figure 1C) with mass effect. Decompressive hemi-craniectomy was performed to relieve the mass effect on the immediate postoperative day but she did not have improvement and died of surgery-related (iatrogenic) ischemic stroke on the 4th postoperative day.

Vasospasm following resection of a skull base tumor is a major insult for concern as it has been associated with a considerable amount of morbidity, mortality, healthcare costs, and disability despite it has not got the attention it has in aneurysmal subarachnoid hemorrhages. An overall incidence of 4.1% was found for vasospasm based on a retrospective analysis of 266 cases. Vasospasms in these cases may have been caused by several preoperative, intraoperative, and postoperative factors that have been identified.

Before adjusting for contributing factors, several factors were found to be associated with vasospasm. These included age, encasement, tumor type (specifically non-meningioma tumors), brain swelling, extent of resection, intracerebral hemorrhage (ICH), subarachnoid hemorrhage (SAH), acute subdural hematoma (ASDH), mass effect, and tumor size. These factors demonstrated a significant association with vasospasm before statistical adjustment.

However, after adjusting for multiple variables using binary logistic regression analysis, the results showed that SAH had a statistically significant association with vasospasm (AOR = 12.27, 95% CI: 1.99–75.37, *P* = .007). This finding suggests that SAH is a strong predictor of vasospasm following skull base tumor resection. Conversely, factors such as high-grade encasement (AOR = 7.03, 95% CI: 0.37–123.49, *P* = .195), non-meningioma tumor type (AOR = 1.37, 95% CI: 0.25–7.53, *P* = .75), and subtotal tumor resection (STR; AOR = 5.09, 95% CI: 0.86–29.89, *P* = .072) did not demonstrate a statistically significant association with vasospasm following adjustment for multiple variables (Table 4).

**Table 4.** Univariate and Multivariate Analysis Results on Factors Associated With Vasospasm in Patients With Skull Base Brain Tumor Resection in a Large Volume Neurosurgical Centers, Ethiopia (*N* = 266)

Item	Variables	Vasospasm		Uni-variate analysis		Multi-variate analysis	
				COR (95%CI)	*P*-value	AOR (95%CI)	*P*-value
		Yes	No				
Age	Below 40	8	136	2.33 (0.61, 8.99)	.218	3.33 (0.54, 20.55)	.194
	above 40	3	119	1		1	
Encasement	Grade 0–2	1	129	1		1	
	Grade 3–5	10	126	10.24 (1.29, 81.15)	.028	7.034(0.37, 134.49)	.195
Tumor	Meningioma	5	177	1		1	
	Non-meningioma	6	78	2.72 (0.8, 9.19)	.106	1.37 (0.25, 7.53)	.715
Intraoperative brain swelling	Yes	2	16	3.32 (0.66, 16.67)	.145	0.938 (0.09, 9.07)	.953
	No	9	239	1		1	
Extent of resection	NTR or GTR	3	168	1		1	
	STR	8	87	5.15 (1.33, 19.90)	.018	5.09 (0.86, 29.89)	.072
ICH	No	6	209	1		1	
	Yes	5	46	3.79 (1.11, 12.94)	.034	1.31 (0.20, 8.44)	.777
SAH	Yes	6	17	16.8 (4.65, 60.71)	.000	12.27 (1.99, 75.37)	.007
	No	5	238	1		1	
ASDH	No	10	249	1		1	
	Yes	1	6	4.15 (0.46, 37.81)	.207	10.81(0.49,37.98)	.131
Mass effect	No	5	201	1		1	
	Yes	6	54	4.46 (1.31, 15.19)	.017	10.22, 8.60)	.743
Size	below 4	1	95	1		1	
	4–6	8	136	5.59 (0.69, 45.42)	.108	3.90 (0.33, 45.11)	.275
	above 6	2	24	7.917(0.69, 0.99)	.097	4.33 (0.25, 75.64)	.315

### Illustrative Case for Cerebral Vasospasm Following Skull Base Tumor Resection

**Figure 2.**
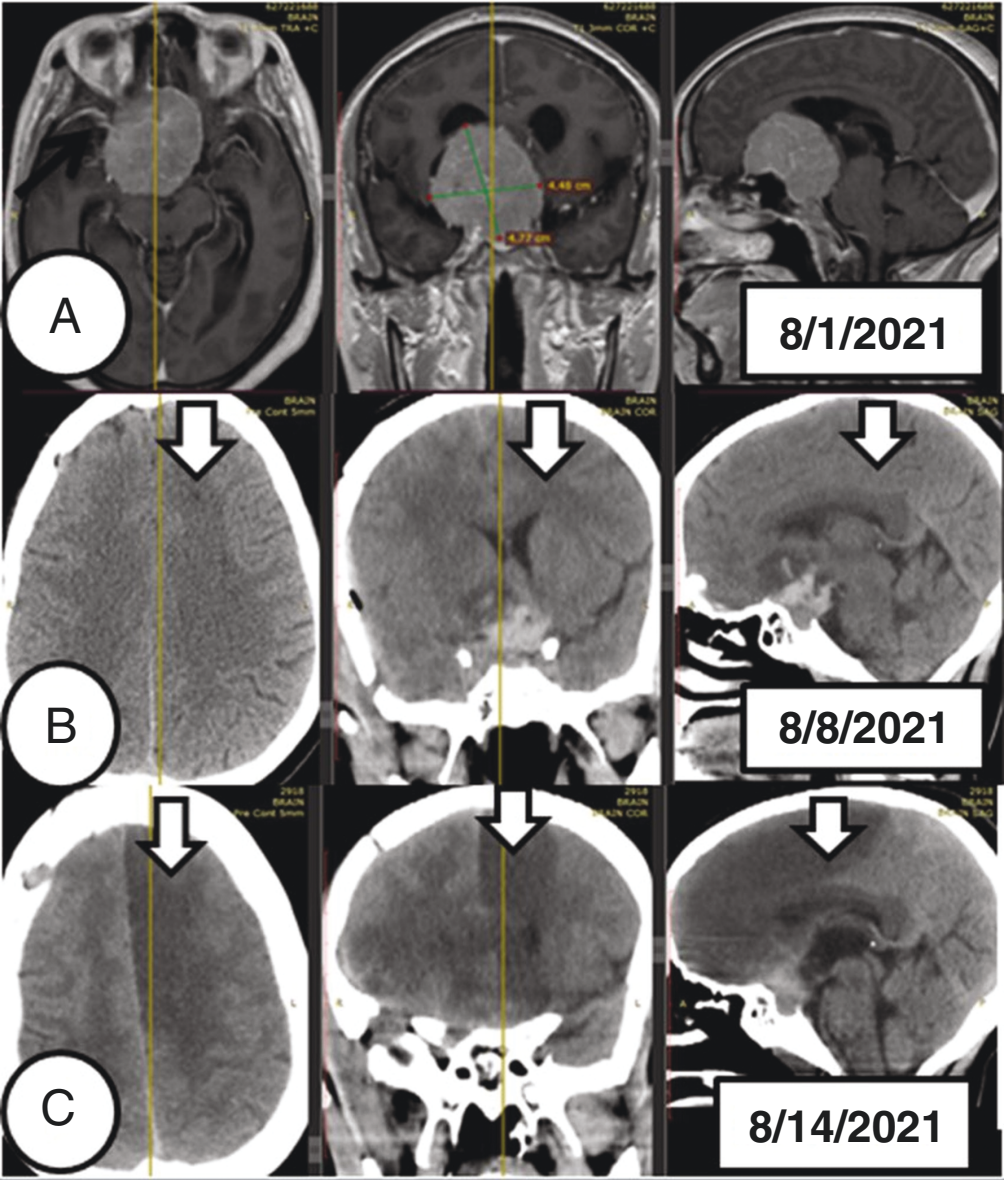
This image depicts the interval radiologic changes in a patient who was diagnosed to have delayed cerebral infarction due to both clinical and radiologic vasospasm after undergoing surgery for tuberculum sella meningioma. The image in (A) showed a giant-size tuberculum sella meningioma partly encasing the right middle cerebral artery. The first postoperative follow-up CT scan in (B) was obtained after 24 hours of the surgery and the down-pointing arrow confirms the absence of iatrogenic vascular injury. The CT in (C) was obtained after 6 days of the first follow-up CT as the patient presented with a new onset of seizure and right-side lower extremity weakness. The down-pointing arrow in (C) depicted left anterior cerebral artery territory infarction secondary to delayed vasospasm.

This was a case of 38 years 38-year-old female patient who presented with a bilateral visual loss of 2 years. Otherwise, she had no prior surgery and comorbidity. Objectively, she had no light perception bilaterally. No other neurologic deficit. Preoperative brain MRI with contrast showed a giant tuberculum sellae meningioma with right MCA encasement (Figure 2A). GTR was achieved with no iatrogenic injury of vessels through right pterional craniotomy. The patient was transferred to ICU and extubated on the first postoperative day. Apart from the visual loss, she had normal neurologic status postoperatively. The follow-up postoperative CT confirmed GTR of the tumor with no evidence of infarction (Figure 2B). She was then discharged on 3rd day with a smooth clinical course. After 3 days of discharge, she presented with a new onset of right-side lower extremity weakness, seizure, and decreased level of consciousness. She was then imaged with a follow-up CT and showed left anterior cerebral artery territory infarction due to delayed cerebral vasospasm (Figure 2C). The patient was readmitted to ICU. The seizure was well controlled with phenytoin while the delayed vasospasm was treated with oral nimodipine and fluid management to achieve euvolemia and augmented hypertension. The weakness continued to worsen over time but the decreased level of consciousness improved after admission. Unfortunately, this patient contracted a COVID-19 infection and died of respiratory failure.

## Discussion

Ischemic cerebrovascular complications following cranial base tumor resection are one of the life-threatening complications. In setups where neurosurgery is yet in its infancy, like most low-income settings including ours, surgery-related ischemic vascular insults in patients undergoing skull base tumors are common (19.9% in this study).^2,19^ In contrast to this, due to the refinement of surgical techniques and the advent of endovascular interventions, it appeared that this complication is extremely rare; especially in better neurosurgical setups like the United States though it still is associated with worse prognosis if it occurs. According to a retrospective review of 155 skull base meningioma patients who underwent surgical resection in the United States, the iatrogenic injury of major and branch vessels turned out to be <1% and <3%, respectively. Similarly, out of the 3 patients who suffered from vascular injury in this cohort, only one patient died, while the remaining 2 patients survived, making the mortality rate 33.3%.^20^

Another US-based surgical center that assessed the incidence of iatrogenic vascular injury among patients who underwent endoscopic skull base surgery showed a 0.5% incidence of iatrogenic injuries out of the 800 patients operated. Endovascular occlusion (for 3 patients) and direct surgical ligation were performed (for 1 case) with a favorable outcome in all cases.^21^

Furthermore, our study revealed that iatrogenic vascular insults and vasospasm accounted for 79.3% and 20.7%, respectively, out of the 53 ischemic vascular complications. The incidence of cerebral vasospasm in our study was 4.1 %, which is 2.2 times more common than the study conducted to assess the incidence of vasospasm, where the incidence was 1.9 % among 470 skull base tumor resections done.^2^ All patients who developed vasospasm in our study were all symptomatic in exact contrast to other studies, where only 1 out 9 patients who developed vasospasm became symptomatic.^2^ This high incidence of vasospasm and overall ischemic cerebrovascular incidents like iatrogenic vessel injury might partly be explained by the larger size tumor (4.9 cm [±IQR 1.5]) and higher encasement grade in our series. The relatively larger tumor size in turn could be attributed to the long average duration of symptoms at the time of presentation, which had a median of 17.3 months (±SD = 11.1). This duration is significantly delayed compared to a study conducted in the United Kingdom, which reported a median duration of 24 days, with a minimum of 7 days and a maximum of 65 days at the time of diagnosis. This degree of discrepancy might be related to poor access to diagnostic neuroimaging services, inadequate knowledge to consider the diagnosis of brain tumors by primary care practitioners, and poor health-seeking behavior of patients in our setting.^22^

The incidence of cerebral vascular infarction in Almefty and Puzzilli et al was 1 out of 28 and 1 out of 33 anterior clinoidal meningioma cases operated, respectively. This means that taking meningioma regardless of location and size, the incidence of vascular insult in our study was found to be 5.25 more common than Almefti’s and Puzzilli et al.^10,23^ Unfortunately enough, 79.3 % (42/53) of patients died of causes related directly or indirectly to ischemic cerebrovascular complications while they were in the hospital due to the absence of chemical and mechanical interventional angioplasty therapeutic options unlike other setups, where they have a better outcome^8^ (Tables 3 and 4).

### Predictors of iatrogenic vascular injury

In skull base tumor resection, there is an inherent risk of vascular injury especially when the tumor’s pathology is fibrous, non-cleavable, invades the surrounding critical neurovascular structures, and surgeons’ operative strategy during an attempt to dissect the tumor off of the vessels.^24^

In our study, female patients had more than 3 times increased risk of iatrogenic injury possibly because female patients are nearly twice as common compared to males in our series, (*P* < .005). This is consistent with most other studies as meningioma is the most common intracranial primary tumor and with female predilection.^1^

Similarly, skull base meningioma was found to have more than 5 times increased risk of iatrogenic injury and this could be related to the inherent propensity of these tumors to encase and even narrow the caliper of the vessels besides the more fibrous nature of these tumors; ultimately making the surgery more difficult and hence increased vessel injury, (*P* < .002). In addition, it could be due to the closer proximity of these tumors (especially clinoidal and tuberculum sella meningioma) to the Circle of Willis.

Furthermore, our study showed that skull base tumor resection through craniotomies was associated with more chance of iatrogenic vascular insult, (*P* < .025). Only 5 (3 vasospasms and 2 iatrogenic injuries) ischemic insults were from trans-sphenoidal approaches. This could be partly attributed to the more fibrous, larger, recurrent, and difficult tumors were dealt with craniotomy than trans-sphenoidal routes. According to a national survey conducted to assess the incidence of carotid artery injury by different neurosurgeons while performing trans-sphenoidal procedures, 958 of them claimed that their injury rate was ranging 1%–2% and they asserted that the trans-sphenoidal route is one of the safer approaches of skull base tumors.^25^ Though there is a lack of comprehensive study on iatrogenic injury while performing craniotomies, the use of real-time Doppler ultrasound was able to reduce the risk of vascular injury to <1% according to the study conducted in 501 patients that underwent craniotomy. This incidence is much lower than the 18% injury incidence while performing skull base tumor resection according to our study. The fact that Doppler solves the imprecision of stereotactic neuro-navigation related to brain shift, its lower cost and reputability to trans-sphenoidal procedures too, its use was recommendable, especially in intricate locations with higher grade vessel encasements as the tumor and vessel could be barely visible.^24^

After adjusting for confounders, patients who had grades 3–5 vessel encasement were statistically associated with iatrogenic injury, AOR [5.04 95% CI (1.79, 14.12)], (*P* < .002). This is in agreement with other studies where tumors having complete vessel encasement (grade 5) were likely to have statistically significant infarction 20% (15/75) compared to lower grade encasement, (*P* < .001). In addition, this study showed that an infarction that developed in patients with higher grade tumors was likely to have a higher infarct volume as evidenced by 12 cc for grade 5 encasement compared to 1.1–9.6 cc for grades 0–4, *P* < .006.^19^ According to this study, the mean infarct volume was 14.6 ± 45.2 cc in exact contrast to our study where the mean infarct volume was 129.6 ± 87.2 cc. This significant difference in the infarct volume could likely be the injury of major vessels in our study.

Perhaps, intimately related to the encasement of vessels is the size of the tumor as larger tumors tend to encase vessels, put a mass effect on the crucial neural structures, and pose technical difficulty in achieving safer dissection and resection. Accordingly, tumor size ≥4cm was likely to have more than 4 times the likelihood of iatrogenic vascular complications compared to smaller tumors, *P* < .002.^19^

Moreover, multivariate binary logistic regression showed that tumors originating from the middle cranial fossa or tumors that extend to both the anterior and middle cranial fossa were associated with more than 6 times increased risk of iatrogenic vessel injury compared to tumors originating purely from anterior cranial fossa, (*P* < .008). This can be explained by the proximity to the circle of Willis and the more technical difficulty of resecting tumors as one gets closer to the central skull base. In addition, it harbors the more common tumors (meningioma) that need more manipulation during resection.^2,19,26–28^

In a multivariate analysis, we found that the adjusted odds of developing iatrogenic vascular insult with attempts made to achieve GTR &/ NTR was, AOR = 2.89, 95% CI (1.01, 8.24), (*P* < .048). This is consistent with another study that showed a statistically significant association between a higher degree extent of resection and surgery-related post-op stroke, (*P* = .041).^9^ This could be due to the repeated attempts to dissect the tumor and maximize the extent of resection besides the possibility of decreased focus that comes from long operative hours and fatigue. In addition, dealing with difficult hemostasis had a statistical association with iatrogenic vessel injury and this might be due to poor visualization of structures and the likely use of overzealous bipolar cauterization in an attempt to arrest bleeding, (*P* < .000).

In addition, intraoperative brain contusion carried higher odds of developing iatrogenic vascular insult, (*P* < .000). This could be explained by attempts to resect the tumor through a narrow operating corridor due to the tight brain that resulted from contusions, poor visualization, and the likelihood of vascular avulsions while dealing with narrow corridors. This underscores the importance of a smooth intraoperative course and holistic preoperative planning.

While prevention of vascular injury should always be the goal, there should also be a well-prepared backup treatment plan to treat iatrogenic vascular injuries in case it happens. Due to the presence of neither the endovascular intervention angio suit nor experienced and trained personnel in revascularising procedures, the treatment of patients with iatrogenic vascular injury in our setup has been suboptimal and associated with a dismal prognosis. In contrast, better neurosurgical centers currently utilize endovascular interventions by making use of digital subtraction angiography.^29,30^

### Vasospasm Predictors

In a separate analysis which was made to look for predictors of vasospasm after adjusting for the confounders, the presence of subarachnoid hemorrhage during and following surgery increased the risk of developing vasospasm 12 times, *P* < .007. This post-tumor resection subarachnoid hemorrhage was found to have a similar vasospasm risk as that of spontaneous aneurysmal rupture subarachnoid hemorrhage. While the exact mechanism of how blood in the subarachnoid space causes cerebral vasospasm is not fully understood, there is little doubt about the vasospasmogenic nature of blood and blood products. Currently, it is accepted that the fresh blood in the subarachnoid space is known to cause early brain injury from transient global ischemia, apoptosis, early metabolic failure, neuro-inflammation, and neuronal dysfunction. Similarly, it is believed that factors involved in the causation of early brain injury partly contribute to the initiation of DCI after 3^rd^ day of the subarachnoid hemorrhage. While a DCI that involves large vessels responds to treatment, it appears that it doesn’t parallel with the neurologic outcome of the patient. Hence, pathophysiological changes after vasospasm of major vessels were considered to have a role and include events like micro-vascular dysfunction, micro-thrombosis, cortical spreading depolarization, and neuro-inflammation; ultimately leading to dysfunction of neurons.^31^

Unlike that for iatrogenic injury, achieving NTR or GTR was shown to have a statistically significant decrease in the occurrence of cerebral vasospasm in a univariate binary logistic regression, OR = 5.15, 95% CI (1.33, 19.90), *P* < .018. This decreased incidence of vasospasm following GTR in our study could be due to a decreased likelihood of postoperative hematomas (SAH and ICH) in patients who underwent GTR/NTR compared to STR; as blood and blood products are widely believed to cause cerebral vasospasm.^31,32^

Furthermore, high-grade (3–5) vessel encasement also showed an increase in vasospasm than those with no or low-grade encasement (0–2) in univariate analysis, *P* < .028. This could be due to more manipulation while trying to dissect the tumor from the vessel because stretch-activated channels allow calcium influx and subsequent vascular smooth muscle contraction and vasospasm occur.^33^

Neither the surgical approach nor the specific corridor used to resect the tumor was significantly associated with the occurrence of vasospasm. This is in contrast to other studies where they showed no vasospasm when the lamina terminalis approach was used compared to resection through optico-carotid and inter-optic corridors (*P* < .05).^34–36^ Similarly, cerebral vasospasm was seen on the side of surgery only in this study. This is not in full agreement with our finding as 2 contralateral artery territories developed vasospasm in our study and bilateral artery involvement was also seen in 2 out of the 11 patients with vasospasm.

While the diagnosis of patients with post-surgery/iatrogenic/ischemic stroke is pretty simple and diagnosed with plain CT/MRI, the diagnosis of cerebral vasospasm and DCI is usually difficult. Accordingly, the American Heart Association /American Stroke Association guideline considers a class II recommendation to screen high-risk patients (eg, patients with aneurysmal subarachnoid hemorrhage) by using trans-cranial Doppler ultrasound and electroencephalography (EEG)^37,38^ given to their less invasiveness and good sensitivity in detection of vasospasm. While the routine use of screening for patients who underwent skull base tumor resection could be controversial, it still might help pick vasospasm at its earlier stage so that delayed cerebral infarction could be prevented. However, there was a lack of these basic diagnostic tools and experienced personnel in our resource-limited setting, and it was after cerebral infarction developed and patients became gravely symptomatic that follow-up image with either CT/MRI was obtained and diagnosed.

In general, there has been a practice consensus to use oral nimodipine 60 mg PO every 4 hours for nearly all skull base tumor resections using a trans-cranial approach. This holds true for a few patients with difficult trans-sphenoidal pituitary tumor resections with the hope of partly preventing (even if evidence does not strongly show the role of nimodipine in preventing vasospasm) and improving neurologic outcome if vasospasm occurs (supported by evidence).^37,38^

Similarly, the treatment of vasospasm has been by using only oral nimodipine in combination with achieving an euvolemic state and augmented hypertension regardless of the grade and severity of the cerebral vasospasm. That means that we have never had the option of either chemical or mechanical angioplasty due to the absence of digital subtraction angiography and angiography suit to perform interventional chemical or mechanical angioplasties for high-grade vasospasms (grades 3 and 4) and stent or flow diversions for major inadvertent cerebrovascular injuries. As a result, patients who had cerebral vasospasm had poor outcomes in our setup. This highlights the multifaceted nature of predictors associated with ischemic cerebrovascular complications and their profound impact on long-term outcomes among patients undergoing skull base tumor resections.

In summary, while prevention of the occurrence of both iatrogenic vascular injury and cerebral vasospasm is the best strategy that should be followed in the resection of skull base tumors by making use of optimal preoperative surgical planning, intraoperative meticulous execution of the preoperative plans and postoperative proactive follow-ups, it is equally recommendable to equip once setup with angio-suits so that endovascular intervention could be given to the needy and unfortunate patients.

### Strength and Limitation

The study looked into ischemic cerebral complications, a very important contributor to morbidity and mortality in skull base surgery, using multi-centric data from the largest neurosurgical centers in our setting. A fairly large sample size of study populations was included in the study and it was unique in its comprehensiveness. The study found clinically important associations that could be used for risk stratification. The study pointed out the major challenges in diagnosing and treating ischemic insults in a low-income setting and boldly pointed out what things to improve. Moreover, the study bravely investigated the problem that is most likely to be experienced in most centers, especially low-income settings. However, it is not without limitations; the retrospective nature of the study contributed to the exclusion of many patients from the study due to an incomplete patient data registry. The diagnostic challenges precluded most of the radiographic vasospasms; which undermines the true incidence of ischemic cerebrovascular complications and poses a challenge towards initiating early treatment. In addition, the absence of endovascular intervention angiography suits in developing settings like ours precluded the attempts made to undergo revascularization procedures or endovascular interventions.

### Ways Forward to Improve

The practice of skull-based neurosurgical services in our setting has had lots of challenges. But, the way forward that skull base neurosurgery has come in low-income settings like Ethiopia still is encouraging and would benefit a lot from improving the following: (1). Practice in a focused and selected area of interest, (2). Get sub-specialty training, (3). Equip the setup with the essential gadgets that maximize the efficacy and safety of the surgery to the patients, (4). Increase availability of diagnostic neuroimaging modalities to detect tumors early, (5). Improve the awareness of patients’ health-seeking behavior early, (6). Advocate policymakers regarding neurosurgical disorder so that adequate fund allocation is possible to fulfill the necessary gadgets, (7) Utilize multimodal treatment strategies like stereotactic radiosurgery, endovascular interventions, interop Doppler ultrasound use, CUSA, neuro-monitoring and soon., (8). Establish a neuro-intensive dedicated ICU, and (9). Refer some of the patients abroad when it is a must and affordable.

## Conclusion

Surgery-related ischemic cerebrovascular complications are common. Thorough perioperative risk stratification and proactive treatment planning are crucial to mitigate vascular insults associated with it. In low-resource settings, neurosurgical services are provided without advanced instruments, leading to more complications. Therefore, it is important to focus on improving neurosurgical setup to enhance patient outcomes.

## Data Availability

Data are available on reasonable request. Availability of data and material: All patients’ clinical and imaging data can be accessed from the corresponding author when needed.
